# Rapid and reliable diagnosis of *Moraxella catarrhalis* infection using loop-mediated isothermal amplification-based testing

**DOI:** 10.3389/fbioe.2023.1330047

**Published:** 2024-01-08

**Authors:** Fei Xiao, Juan Zhou, Xiaolan Huang, Jin Fu, Nan Jia, Chunrong Sun, Zheng Xu, Yi Wang, Lei Yu, Lihui Meng

**Affiliations:** ^1^ Experiment Center, Capital Institute of Pediatrics, Beijing, China; ^2^ Department of Infection Management, Children’s Hospital Affiliated to Capital Institute of Pediatrics, Beijing, China; ^3^ Department of Infectious Diseases, Children’s Hospital Affiliated to Capital Institute of Pediatrics, Beijing, China

**Keywords:** *Moraxella catarrhalis*, loop-mediated isothermal amplification, nanoparticle-based lateral flow biosensor, restriction endonuclease, visual detection

## Abstract

*Moraxella catarrhalis* (*M. catarrhalis*) was an important pathogen closely associated with respiratory tract infections. We employed the loop-mediated isothermal amplification (LAMP) coupled with nanoparticle-based lateral flow biosensor (LFB) and fluorescence testing technique for formulating two diagnostic methods for *M. catarrhalis* detection, termed *M. catarrhalis*-LAMP-LFB assay and *M. catarrhalis*-LAMP-FRT, respectively. The *M. catarrhalis*-LAMP-LFB system incorporated the use of biotin-14-dCTP and a forward loop primer (LF) with a hapten at the 5′ end. This design in LAMP reaction enabled the production of double-labeled products that could be effectively analyzed using the lateral flow biosensor (LFB). For the *M. catarrhalis*-LAMP-FRT assay, the LF was modified with a sequence at 5′ end, and a fluorophore, as well as a black hole quencher, were strategically labeled at the 5′ end and within the middle of the new LF. The restriction endonuclease *Nb.BsrDI* could accurately recognize and cleave the newly synthesized double-strand terminal sequences, resulting in the separation of the fluorophore from the black hole quencher and releasing fluorescence signals. Both assays have been proven to be highly sensitive and specific, capable of detecting genomic DNA of *M. catarrhalis* at concentrations as low as 70 fg, with no cross-reactivity observed with non-*M. catarrhalis* pathogens. Furthermore, both methods successfully identified *M. catarrhalis* in all clinical samples within 1 h that were confirmed positive by real-time PCR, exhibiting superior sensitivity than conventional culture methods. Herein, the newly developed two LAMP-based assays were rapid and reliable for *M. catarrhalis* detection and hold significant promise for deployment in point-of-care (POC) settings.

## Introduction

*Moraxella catarrhalis* (*M. catarrhalis*) has been recognized as an important pathogen of lower respiratory tract infections since the late 1970s (Verghese and Berk, 1991; Verduin et al., 2002). It was commonly associated with chronic lung diseases. The most common clinical syndrome due to *M. catarrhalis* infection is exacerbation of chronic bronchitis in adult populations and acute otitis media among children (Verduin et al., 2002; Morris et al., 2022). In addition, *M. catarrhalis* was also reported to cause several cases of infectious endocarditis (IE) (Ioannou et al., 2022). Moreover, no effective vaccine is currently available for *M. catarrhalis,* and most clinical isolates are resistant to the commonly prescribed antibiotics (Morris et al., 2022; Stubbendieck et al., 2023). Under this context, development of an accurate, rapid and simple laboratory diagnosis method for *M. catarrhalis* detection is especially important for early surveillance, accurate clinical diagnosis, and effective treatment.

Traditional diagnosis methods for *M. catarrhalis* detection mainly included bacterial culture and PCR (polymerase chain reaction)-based methods. Bacterial culture was the most commonly employed method and recognized as the “gold standard” for diagnosis of *M. catarrhalis* infection in clinical settings. However, this method usually required an overnight incubation and a further genus and species level identification, which took more than 48 h, leading to delay of timely diagnosis and effective treatment. Moreover, the similar phenotype between *M. catarrhalis* and *Neisseria* spp. confused the accurate detection of true pathogens (Enright and McKenzie, 1997; Karalus and Campagnari, 2000). PCR-based diagnostic methods were able to provide precise information on the presence or absence of the pathogen of interest with high sensitivity, specificity and rapidness. Within 2 h, PCR-based methods, especially real-time PCR method, were able to achieve accurate diagnosis of *M. catarrhalis* infection with a pair of oligonucleotide primers and a probe (Greiner et al., 2003). PCR-based methods, however, was only carried out in advanced clinical laboratories due to the requirement of expensive equipment and professional technicians, making this method difficult to be popularized in remote areas and point-of-care (POC) testing. Thus, rapid, simple and self-contained diagnostic methods were yet to be developed.

Loop-mediated isothermal amplification (LAMP) is a simple, rapid and self-contained technique for nucleic acid detection (Notomi et al., 2000). Unlike PCR-based methods that relied on complicated instruments, LAMP technique was performed only requiring a simple and cost-effective equipment (such as a heating bath or a cup) that could maintain a constant temperature of 60–65°C (Notomi et al., 2000; Soroka et al., 2021). By using LAMP technology, million-fold amplification products achieved within 15–60 min with two or three pairs of primers and the *Bst* DNA polymerase. Due to the simple amplification procedure and cost-effective instrument, LAMP technology has been utilized for multiple pathogens detection and exhibited extremely high analytical sensitivity and specificity (Zhu et al., 2020; Si et al., 2021; Xiao et al., 2022; Huang et al., 2023). In addition, LAMP products could be analyzed with various formats, including turbidimeters, colorimetric regents, lateral flow biosensor (LFB), fluorescent dyes and more. Particularly, LFB was a more preferable choice for its simplicity, rapidness and cost-effective (Chander et al., 2014). However, most of the previous studies necessitated at least two labeled primers, which complicated the researches.

In this study, we established two LAMP-based methods for simple, rapid and accurate diagnosis of *M. catarrhalis* infection. The two new method, designed by employing LAMP for target amplification and lateral flow biosensor (LFB) or fluorescence detector for result reporting (termed *M. catarrhalis*-LAMP-LFB and *M. catarrhalis*-LAMP-FRT, respectively, and combined as *M. catarrhalis*-LAMP-LFB&FRT), were expected to achieve *M. catarrhalis* detection in POC settings and basic medical facilities in rural areas. The new methods were analyzed for sensitivity and specificity evaluation. Moreover, the usefulness in clinical practice was additionally accessed by clinical samples from respiratory infection patients.

## Materials and methods

### Reagents and instruments

Both common and labeled primers used in this study were synthesized by Aoke BiotechCo., Ltd. (Beijing, China). Visual detection reagent (VDR), biotin-14-dCTP, DNA Isothermal Amplification Kit, and nanoparticle-based lateral flow biosensor (LFB) were provided by Huidexin Biotech Co., Ltd. (Tianjin, China). Restriction endonuclease (*Nb.BsrDI*) was obtained from New England Biolabs Inc. (United States). Genomic DNA kit for nucleic acid extraction and purification was purchased from Beijing Transgen Biotech Co., Ltd. (Beijing, China). Real-time turbidimeter LA-320C was purchased from Eiken Chemical Co., Ltd. (Japan). The BlueSight Pro (GD50502) was purchased from Manod Biotech Co., Ltd. (Suzhou, China).

### Primer design

**FIGURE 1:**
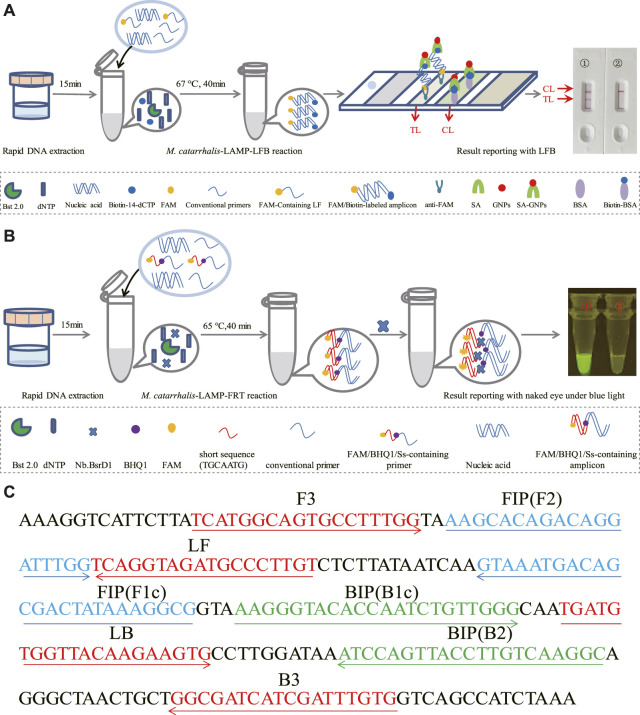
Schematic illustration of the *M. catarrhalis*-LAMP-LFB&FRT assays. **(A)** The detection principle of *M. catarrhalis*-LAMP-LFB assay. In the *M. catarrhalis*-LAMP-LFB system, a FAM-labeled loop primer (LF^#^) and biotin-14-dCTP were utilized, leading to generation of double-labeled amplicons (FAM and biotin labeled), which were captured by the immobilized anti‐FAM of LFB and visualized via reaction between biotin and SA‐GNPs (streptavidin‐coated dyed (crimson red) polymer nanoparticles), resulting in a red color line occurred in the TL region of LFB. The remaining SA‐GNPs were captured by the immobilized biotin‐BSA (biotinylated bovine serum albumin) at the CL region, leading to a red color line occurred in the CL region that indicated the usefulness of the LFB. **(B)** The detection principle of *M. catarrhalis*-LAMP-FRT assay. In the *M. catarrhalis*-LAMP-FRT system, an additional restriction endonuclease *Nb*.*BsrDI* and a modified loop primer (LF*) were utilized. The primer LF* was modified by adding a short sequence (TGCAATG) at 5′ end which could be recognized by restriction endonuclease. *Nb*.*BsrDI* and a fluorophore and a black hole quencher 1 (BHQ1) at the 5′ end and the middle of new primer. When reacted with LF*, the *M. catarrhalis*-LAMP-FRT system generated plenty of Ss-containing target amplicons that were then cleaved by restriction endonuclease *Nb*.*BsrDI*, which could seperate the fluorophore FAM from BHQ1, resulting in emission of fluorescence signals that could be observed by unaided eyes under blue light. **(C)** Sequences and locations of primers used in this study. Right arrows and left arrows indicate sense and complementary sequences that were used.

LAMP enabled highly specific for target sequence amplification for its mechanism of primer design. For the LAMP technology, the primers were designed to recognized the target sequence with six independent sequences (Notomi et al., 2000). Based on this principle, a set of 6 primers spanning 6 independent regions of the target sequence, including two outer primers (F3 and B3), two inner primers (FIP and BIP) and two loop primers (LF and LB), were designed targeting the *copB* gene (Accession no. U69982) of *M. catarrhalis* using Primer Premier 5.0. In order to further ensure the specificity of LAMP reaction, the obtained primer sequences were then analyzed using NCBI Primer-Blast. The primers that nonspecifically matched with other microorganisms were excluded, and the optimal ones were achieved. Of note, the loop primer LF used in *M. catarrhalis*-LAMP-LFB assay (termed LF^#^) was modified by assigning a fluorophore (FAM) at the 5′ end, while that employed in the *M. catarrhalis*-LAMP-FRT assay (termed LF*) was modified by additionally adding a short sequence (Ss, TGCAATG) at the 5′ end and assigning a fluorophore and a black hole quencher 1 (BHQ1) at the 5’ end and the middle of new primer. Sequences, locations and modifications of the primers used in this report were shown in Figure 1; Supplementary Table S1.

### DNA preparation

Genomic DNA of pure culture and clinical samples were extracted using the commercially available EasyPure Bacteria Genomic DNA kit (Beijing Transgen Biotech Co., Ltd.), which was a solid-phase extraction method applicable in POC settings. After extracting and purifying the nucleic acid following manufacture’s instruction, the resultant genomic DNA were stored at −20°C for further use.

### The standard *M. catarrhalis*-LAMP-LFB assay

The standard *M. catarrhalis*-LAMP-LFB reaction was performed in a 25 μL reaction mixture, containing 12.5 μL of 2 × isothermal reaction buffer, 0.1 μM each of outer primers (F3 and B3), 0.2 μM each of loop primers (LF^#^ and LB), 0.4 μM each of inner primers (FIP and BIP),1.0 μL of *Bst* 2.0 DNA polymerase (8 U), 0.5 μL of biotin-14-dCTP, 1.2 mL of VDR, and 1 μL of genomic DNA of *M. catarrhalis* (5 μL for clinical samples). In addition, 1 µL of DNA template from *Neisseria meningitides*, *Klebsiella pneumoniae*, and *Staphylococcus aureus* were employed as negative controls, and 1 µL of double distilled water was used as blank control. The reaction mixtures were incubated at 63°C for 1 h using the real-time turbidimeter to monitor the amplification process. The results were analyzed by three formats, *i.e.*, real-time turbidimeter, VDR and LFB. Result reporting by LFB was performed by adding 5 µL of LAMP reaction products to the LFB sample pad, followed by adding 100 μL of running buffer (10 μM PBS, pH 7.4, containing 1% Tween 20). Results were interpreted within 2 min, with two red lines in both TL (testing line) and CL (control line) regions indicating a positive result, while only one red line in CL region indicating a negative result.

### The standard *M. catarrhalis*-LAMP-FRT assay

The *M. catarrhalis*-LAMP-FRT assay was conducted in a volume of 25 μL reaction system as well, which included 12.5 μL of 2 × isothermal reaction buffer, 0.1 μM each of outer primers (F3 and B3), 0.2 μM each of loop primers (LF* and LB), 0.4 μM each of inner primers (FIP and BIP), 1.0 μL of Bst 2.0 DNA polymerase (8 U), 1.0 μL of restriction endonuclease *Nb*.*BsrDI*, and 1 μL of genomic DNA of *M. catarrhalis* (5 μL for clinical samples). After reacted at 67 °C for 1h, the resultant products were analyzed by visual inspection with naked eye under blue light (BlueSight Pro). Commonly, release of fluorescence signal indicated a positive result, otherwise indicated a negative result.

### Optimization of the *M. catarrhalis*-LAMP assay

To optimize the performance of both methods for *M. catarrhalis* detection, the optimum reaction temperature is determined by performing *M. catarrhalis*-LAMP reaction at temperatures ranging from 62°C to 69°C with 1°C interval. The reaction process was monitored by using the real-time turbidimeter. The temperature at which the fastest reaction speed was obtained was considered as the optimum one, and was utilized for the following assays. Moreover, assays with different reaction time from 10 min to 40 min (with 10 min interval) were conducted as well to determine the optimal reaction time. The obtained optimum reaction conditions were employed for the following tests.

### Specificity of the *M. catarrhalis*-LAMP-LFB&FRT assays

In order to access the analytical specificity of the *M. catarrhalis*-LAMP-LFB&FRT assays, a total of 28 non-*M. catarrhalis* strains were employed (Supplementary Table S2). The results were analyzed by three visually inspected methods, including VDR, LFB and fluorescence detection under blue light. Each test was conducted at least twice.

### Sensitivity of the *M.catarrhalis*-LAMP-LFB&FRT assays

Genomic DNA of *M. catarrhalis* was ten-fold serially diluted (70pg, 7pg, 700 fg, 70 fg, and 7 fg per microliter) to determine the limit of detection (LoD) of the *M. catarrhalis*-LAMP-LFB&FRT assays. 1 μL each of the serial dilutions was utilized as templates for the reactions, the results were revealed by formats of real-time turbidity, VDR, LFB and fluorescence testing under blue light. Each test were repeated in duplicate. The lowest concentration of genomic DNA of *M.catarrhalis* was regarded as the LoD of the *M. catarrhalis*-LAMP-LFB&FRT assays.

### Clinical feasibility of *M. catarrhalis*-LAMP-LFB&FRT assays

To evaluate the feasibility of the *M. catarrhalis*-LAMP-LFB&FRT assays in clinical settings, a total of 48 sputum samples were collected from the outpatient department of the Children’s Hospital Affiliated to Capital Institute of Pediatrics from April 5 to 30 August 2023. All the samples were tested by *M. catarrhalis*-LAMP-LFB&FRT assays. For comparison, real-time PCR and bacterial culture methods were carried out for *M. catarrhalis* detection simultaneously. All the samples employed in this study has been subject to informed consents signed by the subject’s guardian, and the code of Ethics has been approved by the Ethics Committee of Capital Institute of Pediatrics.

## Results

### The mechanism of *M. catarrhalis*-LAMP-LFB&FRT assays

The reaction mechanism of *M. catarrhalis*-LAMP-LFB assay was shown in Figure 1A, which integrated LAMP reaction with LFB detection for *M. catarrhalis* diagnosis. In the *M. catarrhalis*-LAMP-LFB system, a FAM-labeled loop primer (LF^#^) and biotin-14-dCTP were employed for the generation of double-labeled amplicons (FAM and biotin labeled), which were captured by the immobilized anti‐FAM of LFB and visualized via reaction between biotin and SA‐GNPs (streptavidin‐coated dyed (crimson red) polymer nanoparticles), resulting in a red color line occurred in the TL region of LFB. The remaining SA‐GNPs were captured by the immobilized biotin‐BSA (biotinylated bovine serum albumin) at the CL region, leading to a red color line occurred in the CL region that indicated the usefulness of the LFB.

The reaction mechanism of *M. catarrhalis*-LAMP-FRT assay was illustrated in Figure 1B, which combined LAMP reaction with restriction endonuclease cleavage for fluorescent detection of *M. catarrhalis*. In the *M. catarrhalis*-LAMP-FRT system, an additional restriction endonuclease *Nb*.*BsrDI* and a modified loop primer (LF*) were utilized. The primer LF* differed from the conventional loop primer LF in an additional Ss (TGCAATG) at 5′ end which could be recognized by restriction endonuclease. *Nb*.*BsrDI* and a fluorophore and a black hole quencher 1 (BHQ1) at the 5’ end and the middle of new primer. When reacted with LF*, the *M. catarrhalis*-LAMP-FRT system generated plenty of Ss-containing target amplicons that were then cleaved by restriction endonuclease *Nb*.*BsrDI*, which could seperate the fluorophore FAM from BHQ1, resulting in emission of fluorescence signals that could be observed by unaided eyes under blue light.

### Confirmation of for *M. catarrhalis*-LAMP-LFB&FRT assays

**FIGURE 2:**
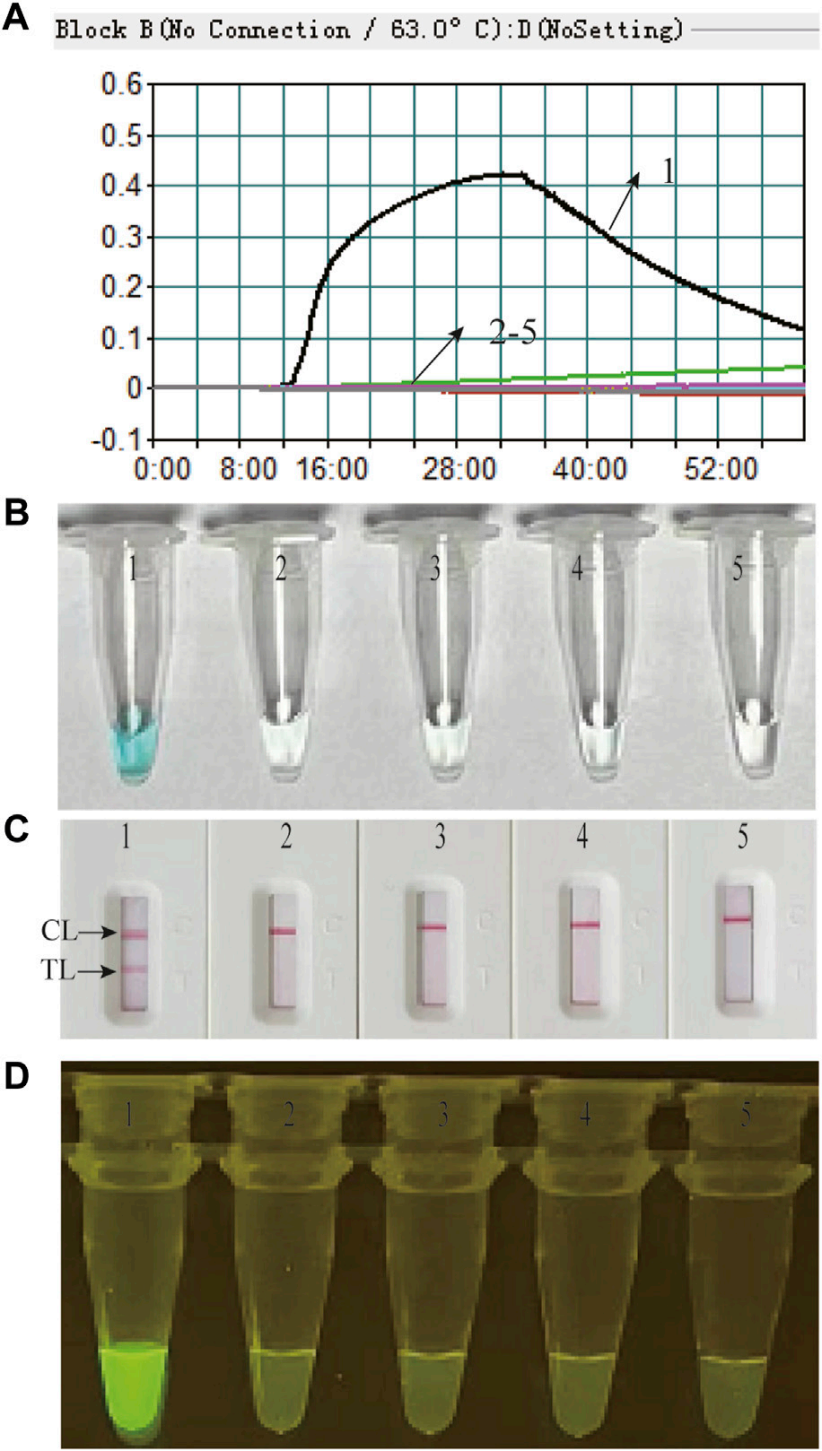
Feasibility of the *M. catarrhalis*-LAMP-LFB assay and *M. catarrhalis*-LAMP-FRT assay for *M. catarrhalis* detection was confirmed by real-time turbidity **(A)**, color change **(B)**, and LFB **(C)** and visual inspection under blue light **(D)** with genomic DNA of *M. catarrhalis* as positive control, that of *Neisseria meningitides*, *Klebsiella pneumoniae*, and *Staphylococcus aureus* as negative control, and distilled water (DW) as blank control. 1–5 indicated reaction results with genomic DNA of *M. catarrhalis*, *Neisseria meningitides*, *Klebsiella pneumoniae*, *Staphylococcus aureus* and DW, respectively. TL, test line; CL, control line.

Feasibility of the *M. catarrhalis*-LAMP-LFB&FRT assays for *M. catarrhalis* diagnosis were confirmed by performing *M. catarrhalis*-LAMP-LFB&FRT reactions in the presence or absence of genomic DNA of *M. catarrhalis* at 63°C for 1 h and using real-time turbidimeter, VDR, LFB and fluorescence detector to analyze the results. Using real-time turbidimeter, a significant increase of turbidity was observed in the reaction with genomic DNA of *M. catarrhalis* (positive control), while the ones with non-*M. catarrhalis* templates (negative controls) or distilled water (blank control) displayed an almost blunt curve (Figure 2A). By using VDR, the color of positive control changed into light green, while the others were colorless (Figure 2B). By using LFB, two visible red lines in the TL and CL regions were observed with products of positive control, while only one line in CL was seen when products of negative controls and blank controls were examined (Figure 2C). Using fluorescence detector, light yellow color releasing by FAM was observed from reaction of positive control, while absence of fluorescence signals was seen in the negative controls and blank controls (Figure 2D). These data demonstrated that the *M. catarrhalis*-LAMP-LFB&FRT assays were applicable for detection of *M. catarrhalis*.

### Optimal reaction condition of the *M. catarrhalis*-LAMP assay

In this report, we performed *M. catarrhalis-*LAMP assay at eight different temperatures ranging from 62°C to 69°C at 1°C intervals for 60 min to obtain the optimum reaction temperature. As shown in Supplementary Figure S1, the reaction incubated at 65–66°C was the fastest one reaching the threshold value of 0.1 of absorbance. In addition, the *M. catarrhalis*-LAMP-LFB&FRT assays were conducted at 65–66°C for 10 min, 20 min, 30 min, and 40 min, respectively, to reveal the optimal reaction time. As shown in Supplementary Figure S2, only after amplified at 65°C for 40 min, the LoD level of genomic DNA of *M. catarrhalis* (determined in the sensitivity analysis) could be detected. Thus, a reaction temperature of 65°C and a reaction time of 40 min were employed in the following tests.

### Sensitivity of the *M. catarrhalis*-LAMP-LFB&FRT assays

**FIGURE 3:**
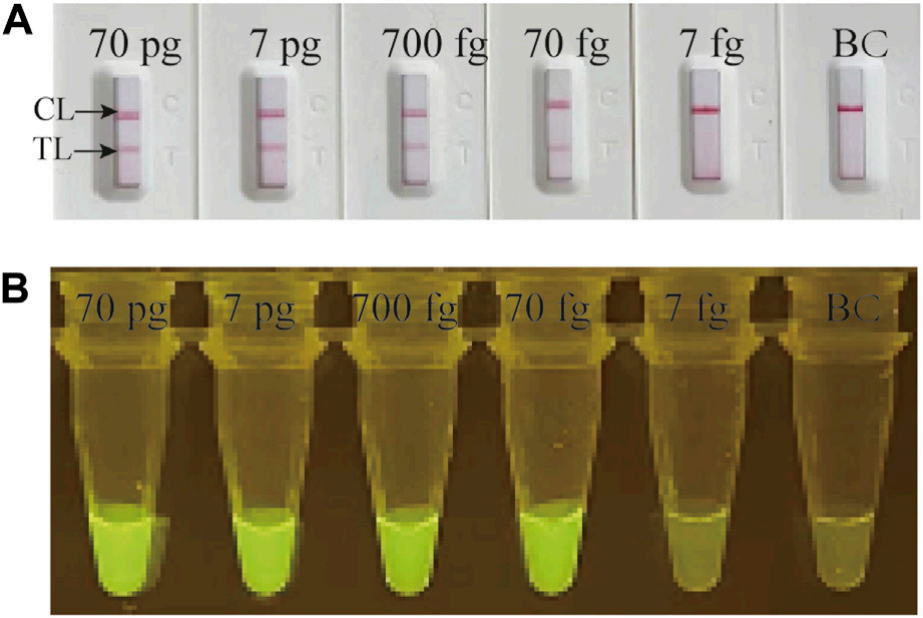
Analytical sensitivity evaluation of *M. catarrhalis*-LAMP-LFB&FRT assays Serial dilutions of genomic DNA of *M. catarrhalis* (from 70 pg to 7 fg per microliter) were employed to evaluation the analytical sensitivity of *M. catarrhalis*-LAMP-LFB assay **(A)** and *M. catarrhalis*-LAMP-FRT assay **(B)**. TL, test line; CL, control line.

Serial dilution of genomic DNA of *M. catarrhalis* were utilized to determine the LoD of the *M. catarrhalis*-LAMP-LFB&FRT assays. As shown in Figure 3, by LFB, the *M. catarrhalis*-LAMP-LFB assay was able to detect down to 70 fg (∼33 copies) of genomic DNA of *M. catarrhalis* per reaction (Figure 3A). Similarly, using fluorescence detector, the lowest concentration of genomic DNA of *M. catarrhalis* detected by the *M. catarrhalis*-LAMP-FRT assay was 70 fg (∼33 copies) as well (Figure 3B). Hence, the LoD of the *M. catarrhalis*-LAMP-LFB&FRT assays were both70 fg (∼33 copies) per reaction, which were in accordance with those indicated by real-time turbidity (Supplementary Figure S3A) and VDR (Supplementary Figure S3B).

### Specificity of the *M. catarrhalis*-LAMP-LFB&FRT assays

**FIGURE 4:**
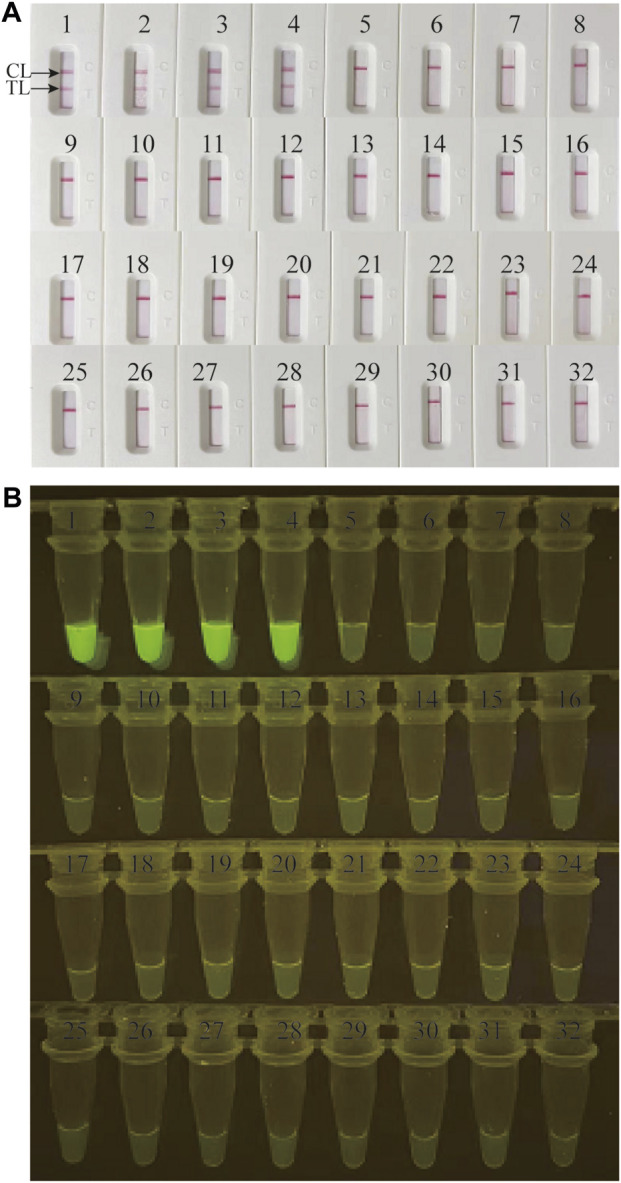
Analytical specificity of *M. catarrhalis*-LAMP-LFB&FRT assays. The specificity of *M. catarrhalis*-LAMP-LFB assay **(A)** and *M. catarrhalis*-LAMP-FRT assay **(B)** were analyzed using 28 non-*M. catarrhalis* pathogens and 4 *M. catarrhalis* strains. 1-4 represented the 4 *M. catarrhalis* strains; 5–32 represented the 28 non-*M. catarrhalis* pathogens (Supplementary Table S2). TL, test line; CL, control line.

Genomic DNA templates of 28 non-*M. catarrhalis* pathogens and 4 *M. catarrhalis* strains were utilized in this study to estimate the specificity of the *M. catarrhalis*-LAMP-LFB&FRT assays. When monitored by LFB, all the 28 non-*M. catarrhalis* pathogens displayed only one red line in CL of LFB, which were totally different from that of the 4 *M. catarrhalis* strains that two red lines were seen at both TL and CL of LFB (Figure 4A). When analyzed using fluorescence detector, light yellow color was only observed from products of 4 *M. catarrhalis* strains rather than those of the 28 non-*M. catarrhalis* pathogen (Figure 4B). Those results suggested that the specificity of the *M.catarrhalis*-LAMP-LFB&FRT assays were 100%.

### Clinical feasibility verification of *M. catarrhalis*-LAMP-LFB&FRT assays

**FIGURE 5:**
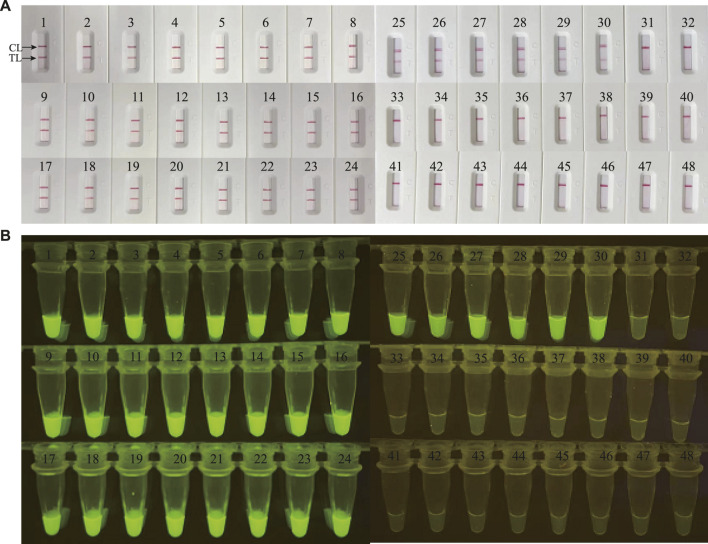
Clinical validation of *M. catarrhalis*-LAMP-LFB&FRT assays with clinical specimens. A total of 48 clinical samples were tested by *M. catarrhalis*-LAMP-LFB assay **(A)** and *M. catarrhalis*-LAMP-FRT assay **(B)** to confirm the clinical feasibility. TL, test line; CL, control line.

**FIGURE 6:**
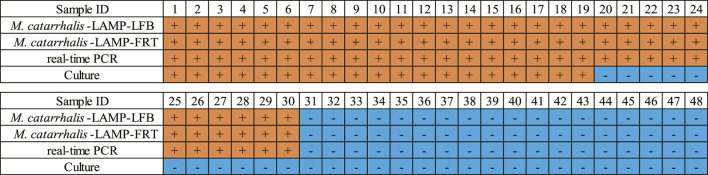
Clinical performance comparison of the *M. catarrhalis*-LAMP-LFB&FRT assays, real-time PCR and culture methods. +, positive result; -, negative result.

In order to validate the clinical feasibility of the *M. catarrhalis*-LAMP-LFB&FRT assays, a total of 48 sputum samples from patients suspected of respiratory infection were simultaneously detected by *M. catarrhalis*-LAMP-LFB&FRT assays, real-time PCR and bacterial culture methods. Of the 48 samples, 30 were tested positive by the *M. catarrhalis*-LAMP-LFB&FRT assays and real-time PCR methods, only 19 samples were confirmed positive by bacterial culture methods (Figures 5, 6). Moreover, 18 samples were tested negative by all the four methods mentioned above. These results demonstrated that the clinical performance of the newly developed *M. catarrhalis*-LAMP-LFB&FRT assays was comparable to that of real-time PCR method but superior to the culture method. Hence, the newly developed *M. catarrhalis*-LAMP-LFB&FRT assays could be used as advanced technology to diagnose *M. catarrhalis* infection in clinical settings.

## Discussion

To date, *M. catarrhalis* has been considered as an important respiratory pathogen in human beings. However, prevention and controlling *M. catarrhalis* infection faced the challenges of high versatility of pathogenicity, high incidence of β-lactam resistance, as well as inadequate effective vaccine. Under this context, rapid, sensitive and accurate detection methods for *M. catarrhalis* were vitally crucial. Although bacterial culture and PCR-based methods were able to accurately diagnose *M. catarrhalis* infection, their shortcomings, including long turn-around time, requirement of expensive instruments and professional technicians, hampered the application in rural areas and field and POC settings. In this context, we developed two LAMP-based diagnostic systems (termed *M. catarrhalis*-LAMP-LFB&FRT) to achieve timely, simple and accurate detection of *M. catarrhalis* in these resource-limited settings.

The new *M. catarrhalis*-LAMP-LFB&FRT assays were devised by combining LAMP amplification with visual inspection of results via LFB and fluorescence detector, resulting in the detection of *M. catarrhalis* time-effective and easy-to-operation. Since LAMP technology was proposed by Notomi et al., in 2000 (Notomi et al., 2000), LAMP technique has been modified into diverse formats for microorganism detection due to its inherent advantages (Wang et al., 2015; Wong et al., 2018; Xiao et al., 2022). LAMP was an isothermal nucleic acid amplification technique that could achieved targets millions-fold increased only by incubated in a heating bath for 30–40 min. The four (or six) different primers used in LAMP reaction ensured the high specificity of the amplification products (Notomi et al., 2000). Although products of LAMP reaction were able to be directly read via changes of turbidity value, various detection techniques, including VDR, LFB, fluorescence detector, and more (Wong et al., 2018), have been applied to improve the accuracy and sensitivity of LAMP-based diagnostic system. Different formats own respective characterizes and mechanisms, such as the turbidimetry method required the specialized instrument (Realtime Turbidimeter) and achieved positive results on the basis of turbidity increase, and the VDR method reported a positive result based on combination of dyes with double-strand nucleic acid, both of which were not specific enough for target characterization. In this study, with a modified primer (LF^#^) and biotin-14-dCTP, products of LAMP reaction was able to be detected by LFB; with an additional restriction endonuclease *Nb*.*BsrDI* and another kind of modification of primer LF (LF*), fluorescent detection of LAMP reaction products was achieved. Different from the previous LFB-based techniques, the LFB-based detection method developed here employs only a hapten-labeled primer and biotin-14-dCTP rather than two hapten-labeled primers, which simplified experimental design and decreased experimental cost. Both detection formats enabled reporting results by unaided eyes rather than complicated equipment, leading to *M. catarrhalis* detection more accessible for everyone and in everywhere. Comparatively, the *M. catarrhalis*-LAMP-FRT assay exhibited superiority to the *M. catarrhalis*-LAMP-LFB assay in no aerosol pollution generation for its unnecessary to open reaction tube, but was limited in the dependence of fluorescent detector rather than visually inspection. The optimized *M. catarrhalis*-LAMP-LFB&FRT assays could accomplish *M. catarrhalis* detection within an hour, resulting in timely treatment and control of *M. catarrhalis* infection. Hence, the *M. catarrhalis*-LAMP-LFB&FRT assays developed here were preferred methods of early diagnosis and treatment of *M. catarrhalis* infection.

The newly developed *M. catarrhalis*-LAMP-LFB&FRT assays exhibited extreme sensitivity and specificity in *M. catarrhalis* detection. The LoD value of both new methods were 70 fg of genomic DNA of *M. catarrhalis*. This level was slight higher than that of real-time PCR method (Greiner et al., 2003) and the electrochemical biosensor method (Sande et al., 2023). Moreover, none of the *M. catarrhalis*-LAMP-LFB&FRT assays cross-reacted with non- *M. catarrhalis* pathogen, indicating a specificity of 100%. The analytical sensitivity and specificity enabled *M. catarrhalis*-LAMP-LFB&FRT assays great potential in accurate and reliable diagnosis of *M. catarrhalis* infection.

Rapid, sensitive and accurate detection of the causative agent of disease played a critical role in the guidance for efficient treatment in clinical settings. The *M. catarrhalis*-LAMP-LFB&FRT assays display excellent performance in clinical practice. In terms of the 48 suspicious specimens, 30 (62.5%) were tested positive by the two methods, which was identical to that by real-time PCR method (30, 62.5%), but obviously superior to that by culture method (19, 39.6%). Although no significant statistical difference was observed between the new methods and real-time PCR method, it would be further validated if more clinical samples were involved. The detection efficiency of *M. catarrhalis*-LAMP-LFB&FRT assays was significantly higher than that of culture method (39.6%), which was in accordance with the previous reports (Si et al., 2021). What’s more, the newly developed *M. catarrhalis*-LAMP-LFB&FRT assays enabled short turnaround time of less than 1 h for *M. catarrhalis* detection along with highly specific and efficient, further implying the great application potential of the newly developed methods in clinical diagnostics.

In conclusion, in this study we successfully established the *M. catarrhalis*-LAMP-LFB&FRT assays, which employed metal or water bath for heating and LFB or fluorescence for reporting LAMP assays. The two new methods enabled timely, simple and reliable detection of *M. catarrhalis* within 1 h. Results of analytical sensitivity and specificity as well as its clinical feasibility performance demonstrated that the *M. catarrhalis*-LAMP-LFB&FRT assays were sensitive, specific and versatile for *M. catarrhalis* detection, highlighting their extensive application in various settings including clinics, resource-limited areas and POC settings. However, it was important to note that the risk of carryover contamination may increased when large scale tests of *M. catarrhalis*-LAMP-LFB were performed in the same lab, resulting in false positive results, thus careful prevention measures should be taken regularly.

## Data Availability

The original contributions presented in the study are included in the article/Supplementary Material, further inquiries can be directed to the corresponding authors.
