# Supplementary material for: Rapid and reliable diagnosis of Moraxella catarrhalis infection using loop-mediated isothermal amplification-based testing
Source: Front Bioeng Biotechnol. 2024 Jan 8;11:1330047. doi: 10.3389/fbioe.2023.1330047 (PMC10800902; doi:10.3389/fbioe.2023.1330047)
Supplement: Supplementary file 1 [file DataSheet1.docx]

**Rapid and reliable diagnosis of *Moraxella catarrhalis* infection using loop-mediated isothermal amplification-based testing**

Fei Xiao ^1,†^, Juan Zhou ^1,†^, Xiaolan Huang ^1^, Jin Fu ^1^, Nan Jia ^1^, Chunrong Sun ^1^, Zheng Xu ^1^, Yi Wang ^1,*^, Lei Yu ^2,*^, Lihui Meng ^3,*^

^1^ Experiment Center, Capital Institute of pediatrics, Beijing, China

^2^ Department of Infection Management, Children's Hospital Affiliated to Capital Institute of Pediatrics, Beijing, 100020, China

^3^ Department of Infectious Diseases, Children's Hospital Affiliated to Capital Institute of Pediatrics, Beijing 100020, China

^†^ These authors contributed equally to this article.

^*^ Correspondence:

Yi Wang: [wildwolf0101@163.com](mailto:wildwolf0101@163.com)

Lei Yu: [15210301056@163.com](mailto:15210301056@163.com)

Lihui Meng: [mengmlh@163.com](mailto:mengmlh@163.com)

**Tables**

**Table S1 Sequences and modifications of the primers used in this study**

| Primers^a^ | Sequence (5’-3’) and and modifications | Length^b^ |
| --- | --- | --- |
| F3 | TCATGGCAGTGCCTTTGG | 18 nt |
| B3 | CACAAATCGATGATCGCC | 18 nt |
| FIP | CGCCTTTATAGTCGCTGTCATTTAC-AAGCACAGACAGGATTTGG | 45mer |
| BIP | AAGGGTACACCAATCTGTTGGG-GCCTTGACAAGGTAACTGGAT | 44mer |
| LF | ACAAGGGCATCTACCTGA | 18nt |
| LB | TGATGTGGTTACAAGAAGTG | 20 nt |
| LF^#^ | FAM-ACAAGGGCATCTACCTGA | 18 nt |
| LF* | FAM-TGCAATG-ACAAGGGCAT(BHQ1) CTACCTGA | 18 nt |

^a^F3, forward outer primer; FIP, forward inner primer; LF, Forward loop primer; LB, Backward loop primer; LF*, 5’-labeled with FAM and BHQ1 when used in *M. catarrhalis*-LAMP Flu assay; LF^#^, 5’-labeled with FAM when used in *M. catarrhalis*-LAMP-LFB assay. BIP, backward inner primer; B3, backward outer primer;

^b^nt, nucleotide; mer, monomeric unit.

**Table S2 Strains used in this study**

| **Pathogen** | **Strain no. (source of strains)^a^** | **No. of strains** |
| --- | --- | --- |
| *Moraxella catarrhalis* | Isolated strains (CIP) | 4 |
| *Acinetobacter baumannii* | Isolated strains (CIP) | 1 |
| *Haemophilus influenzae* | Isolated strains (CIP) | 1 |
| *Pseudomonas aeruginosa* | Isolated strains (CDC) | 1 |
| *Monilia albican* | Isolated strains (CDC) | 1 |
| *Citrobacter spp.* | Isolated strains (CDC) | 1 |
| *Streptococcus pneumoniae* | Isolated strains (CDC) | 1 |
| *Mycobacterium tuberculosis* | Isolated strains (CDC) | 1 |
| *Escherichia* *coli* | Isolated strains (CDC) | 1 |
| *Enterococcus faecalis* | Isolated strains(CDC) | 1 |
| *Klebsiella pneumoniae* | Isolated strains (CDC) | 2 |
| *Listeria innocua* | Isolated strains (CDC) | 1 |
| *Listeria monocytogenes* | Isolated strains (CDC) | 1 |
| *Corynebacterium sriatum* | Isolated strains (CDC) | 1 |
| *Streptococcus aureus* | Isolated strains (CDC) | 1 |
| *Neisseria meningitidis* | Isolated strains (CDC) | 2 |
| *Nocardia asteroides* | Isolated strains (CDC) | 1 |
| *Bacillus cereus* | Isolated strains (CDC) | 1 |
| *Streptococcus pyogenes* | Isolated strains (CDC) | 1 |
| *Salmonella* spp. | Isolated strains (CDC) | 1 |
| *Shigella baumannii* | Isolated strains (CDC) | 1 |
| *Shigella sonnei* | Isolated strains (CDC) | 1 |
| *Staphylococcus amber* | Isolated strains (CDC) | 1 |
| *Staphylococcus epidermidis* | Isolated strains (CDC) | 1 |
| *Staphylococcus haemolyticus* | Isolated strains (CDC) | 1 |
| *Stenotrophomonas maltophilia* | Isolated strains (CDC) | 1 |
| *Steptococcus salivarius* | Isolated strains (CDC) | 1 |

**^a^**CIP, Capital Institute of Pediatrics; CDC, Chinese Center for Disease Control and prevention.

**^b^**P, positive; N, negative.

**Figures**


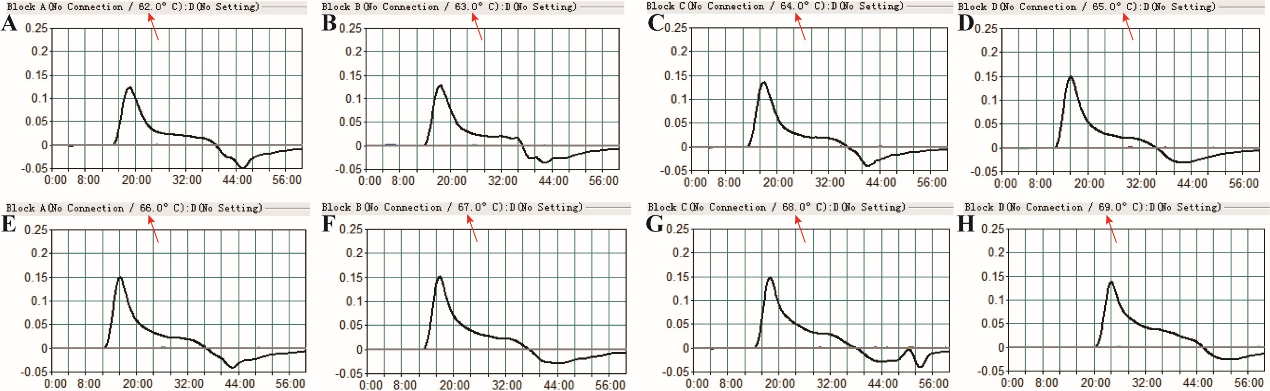


**Figure S1**. **Optimization of reaction temperature of *M. catarrhalis*-LAMP assay.**

The *M. catarrhalis*-LAMP assay were carried out at 62-69 °C (**A-H**) for 1h to determine the optimum reaction temperature. Turbidity > 0.1 was considered positive.


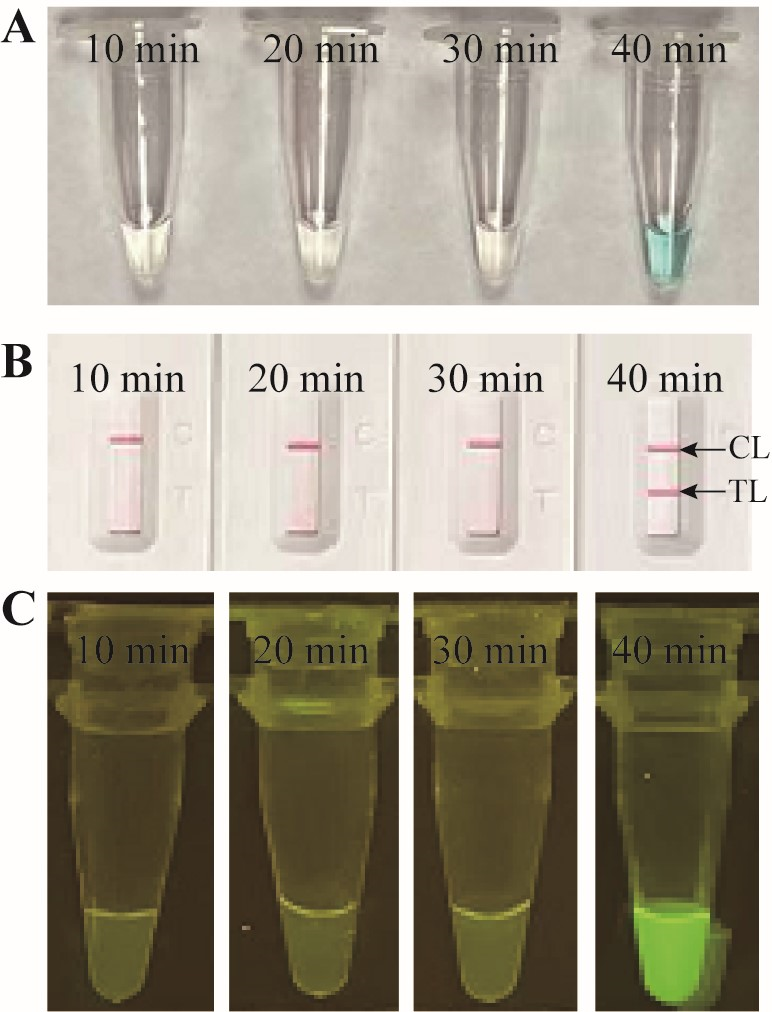


**Figure S2. Optimization of reaction temperature of *M. catarrhalis*-LAMP-LFB&Flu assays.**

The *M. catarrhalis*-LAMP-LFB&FRT assays were performed for 10 min, 20 min, 30 min and 40 min respectively to obtain the optimum reaction. Results were indicated using three methods, *i.e.* colorimetric indicator (**A**), LFB (**B**) and fluorescence detector with by naked eye. TL, test line; CL, control line.


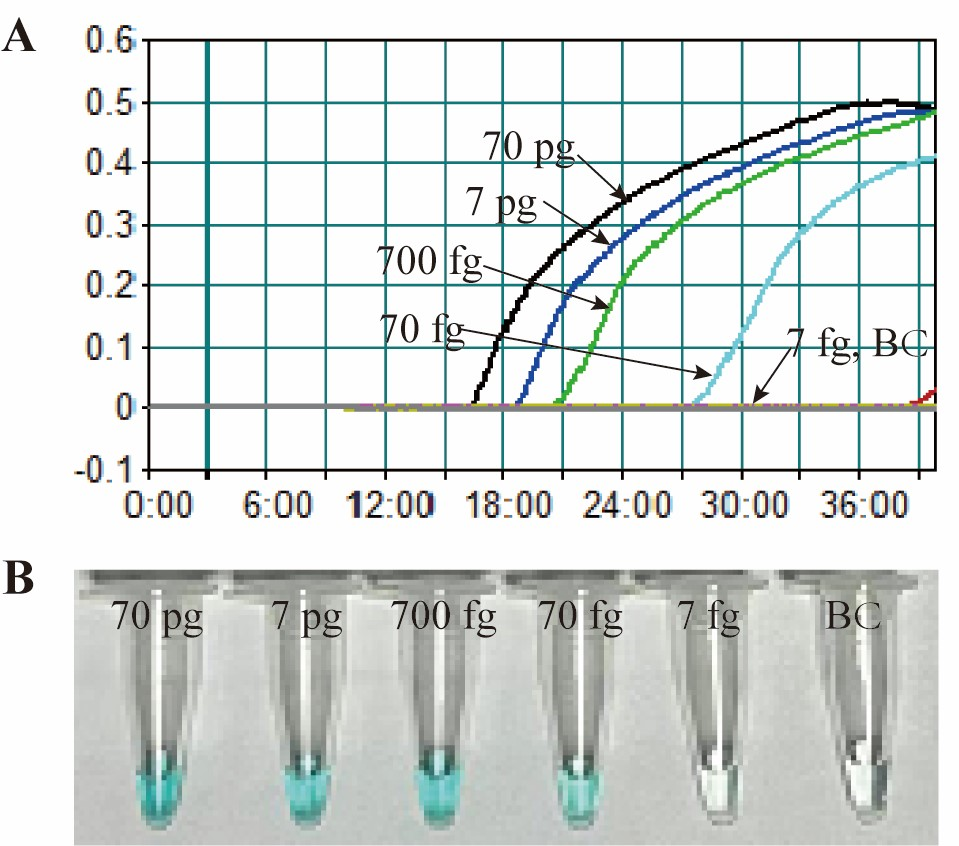


**Figure S3. Sensitivity of *M. catarrhalis*-LAMP assays.**

Serial dilutions of genomic DNA of *M. catarrhalis* (from 70 pg to 7 fg per microliter) were employed to evaluation the sensitivity of *M. catarrhalis*-LAMP assay. The results were displayed by two formats, namely, real-time turbidity (**A**) and colorimetric indicator (**B**).
